# Quality of Narratives in Assessment: Piloting a List of Evidence-Based Quality Indicators

**DOI:** 10.5334/pme.925

**Published:** 2023-05-26

**Authors:** Molk Chakroun, Vincent R. Dion, Kathleen Ouellet, Ann Graillon, Valérie Désilets, Marianne Xhignesse, Christina St-Onge

**Affiliations:** 1 Faculty of medicine and health sciences, Universitéde Sherbrooke, Sherbrooke, Québec, CA; 2 Paul Grand’Maison de la Sociétédes médecins de l’Universitéde Sherbrooke research chair in medical education, Sherbrooke, Québec, CA; 3 Centre de pédagogie et des sciences de la santé, Faculty of medicine and health sciences, Universitéde Sherbrooke, Sherbrooke, Québec, CA; 4 Department of Pediatrics, Faculty of medicine and health sciences, Universitéde Sherbrooke, Sherbrooke, Québec, CA; 5 Department of Family and Emergency Medicine, Faculty of medicine and health sciences, Universitéde Sherbrooke, Sherbrooke, Québec, CA; 6 Department of Medicine, Faculty of medicine and health sciences, Universitéde Sherbrooke, Paul Grand’Maison de la Sociétédes médecins de l’Universitéde Sherbrooke research chair in medical education, Sherbrooke, Québec, CA

## Abstract

**Background & Need for Innovation:**

Appraising the quality of narratives used in assessment is challenging for educators and administrators. Although some quality indicators for writing narratives exist in the literature, they remain context specific and not always sufficiently operational to be easily used. Creating a tool that gathers applicable quality indicators and ensuring its standardized use would equip assessors to appraise the quality of narratives.

**Steps taken for Development and Implementation of innovation:**

We used DeVellis’ framework to develop a checklist of evidence-informed indicators for quality narratives. Two team members independently piloted the checklist using four series of narratives coming from three different sources. After each series, team members documented their agreement and achieved a consensus. We calculated frequencies of occurrence for each quality indicator as well as the interrater agreement to assess the standardized application of the checklist.

**Outcomes of Innovation:**

We identified seven quality indicators and applied them on narratives. Frequencies of quality indicators ranged from 0% to 100%. Interrater agreement ranged from 88.7% to 100% for the four series.

**Critical Reflection:**

Although we were able to achieve a standardized application of a list of quality indicators for narratives used in health sciences education, it does not exclude the fact that users would need training to be able to write good quality narratives. We also noted that some quality indicators were less frequent than others and we suggested a few reflections on this.

## Background & Need for Innovation

Along with the implementation of competency-based medical education (CBME), there has been a recent call for the qualitative appreciation of students’ performance [1,2]. As such, narratives are increasingly used in health sciences education (HSE) [3,4]. A narrative is a form of qualitative assessment that contains written comments, a set of sentences, or a set of words. It is a description, provided by an assessor, of a student’s performance on a specific task in a given context [5]. The use of narratives can serve many purposes in assessment. For example, they can be used in a formative setting to provide information to the learner regarding gaps that need to be corrected or they can be used to monitor learning [3,6]. Furthermore, narratives can serve to inform committee summative decisions about learners’ progression toward competence [3,7]. Since “A decision is only as good as the data on which it was founded” [8], narratives used for the assessment of learners should meet high quality standards [2,3,8,9,10].

Unfortunately, narratives often seem to lack quality [6,7,11]. For example, narratives tend to include ambiguous statements that can be interpreted by learners without grasping the nuances that may be hidden between the lines [6,11,12]. At face value, narratives may convey positive messages, but have codes that must be deciphered by the learner to understand their meanings [6]. Another limitation to the quality of narratives is when assessors only provide positive comments for reinforcement and put aside constructive comments [7]. Such cases induce noise in assessment data which, hinder learners’ ability to identify areas needing remediation [7]. All of these markers of poor quality narratives can negatively influence the validity of the interpretation of assessment data and consequently, the validity of decisions [7,9].

Some quality indicators for writing narratives exist in the literature, but they remain context specific and not always sufficiently operational to be easily used. In the context of clinical competency evaluation, Dudek and colleagues [13] developed a tool that aims at helping supervisors to not only complete, but also assess, the quality of In-training evaluation reports (ITERs). Although validated, the “Completed Clinical Evaluation Report Rating (CCERR)” remains specific to clinical evaluation reports. More recently, Kelly and colleagues [14] developed a tool called “The Narrative Evaluation Quality Instrument”. This tool was created to assess “three specific components within clerkship assessment: performance domains, specificity, and usefulness to learner”. While recognizing the important contribution of this work, enabling the quality monitoring of narrative assessments at the clerkship level, we note that this initiative does not allow a complete portrait of the quality indicators that a narrative performance assessment should present. The “Quality improvement instrument” is another initiative to measure the quality of written feedback in the context of workplace-based assessment. Although it seems to allow for improvement in terms of quality of feedback, the conception of good quality feedback seems a little narrow since the tool only includes two aspects: the strengths and the areas for improvement. More recently, Ross et al. [15] developed an evidence-based tool to evaluate the quality of written feedback. They produced an easy-to-use tool to evaluate narratives intended for residents and unsurprisingly, it has a “clinical color”, that does not allow for generalisability in other contexts. Providing assessors at the undergraduate medical education level (UGME) and administrators with a checklist of quality indicators for narratives could help them more rigorously evaluate the quality of the data used to make decisions about learners.

### Goal of Innovation

Our aim was to create and pilot a list of evidence-based quality indicators comprised of quality indicators previously identified through a rigorous scoping review [10]. This tool was meant to be operational and user-friendly. We also wanted its application to be able to document the quality of narratives used in different contexts. Therefore, we aimed to verify its potential for a standardized application.

## Steps taken for Development and Implementation of innovation

### 1-Creation of a tool of quality indicators for narratives

We used DeVellis’s [16] 8-step framework for scale development to create a tool of quality indicators for narratives used in HSE [3,4].

**Figure 1:**
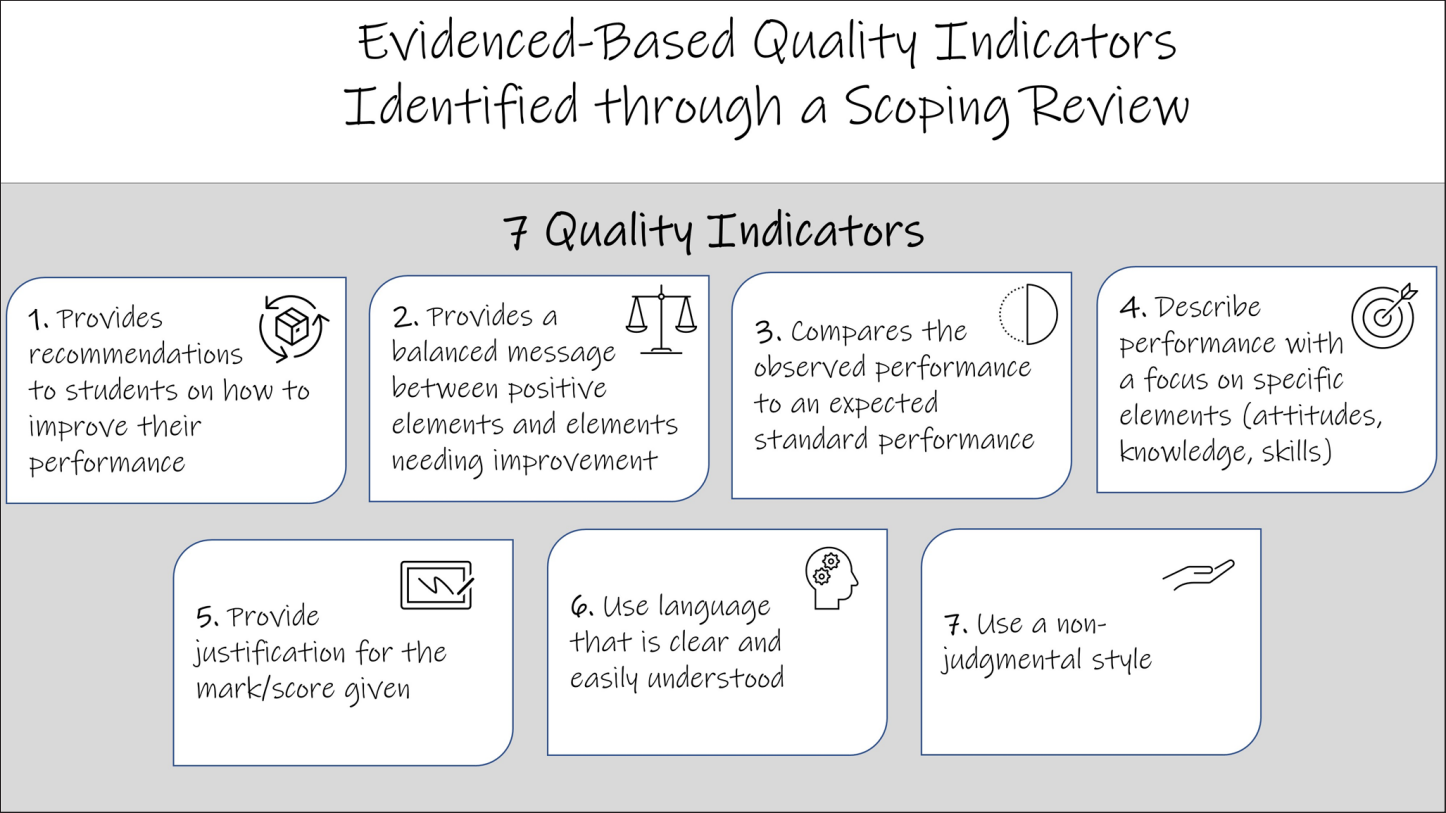
Evidence-based quality indicators of narratives.

Steps 1 (*define the quality of narratives*) and 2 (*generate items*) were completed in a recent scoping review [10]. A main result of the review was the identification of seven quality indicators which are presented in Figure 1. These indicators are the result of the analysis of 47 articles that included trainee and assessor perspectives. We intended to identify indicators to assess the quality of written formative feedback comments that could also be used in the context of summative assessments. We identified indicators that were not specific to a training level to be able to use the tool in either to UGME or postgraduate medical education (PGME) contexts.

We determined that a checklist format (*step 3: determine the format of measurement*) would be a user-friendly approach for this tool [16]. Binary items are easy to answer and generally preferred by individuals versus other formats requiring higher concentration and more judgement [16].

In Step 4 (*have initial item pool reviewed by experts*) we solicited input on item pool of indicators from three potential users and three researchers in HSE. They were asked to assess the clarity and the pertinence of each indicator using a 10-point Likert scale. Furthermore, they were asked to provide suggestions for reformulation and improvement when applicable. After receiving their feedback, the core team members (MC & KO & VRD & CS-O) met, discussed, and applied minor changes.

Steps 5 of DeVellis [16] (*inclusion of validation items*) was not carried out in this study. Step 5 suggests the inclusion of additional items in studies where there is a sensitive construct or a vulnerable population which is not applicable to this study.

### 2- Verifying the potential of the tool for a standardized application

Once the checklist created, we piloted it with a sample of narratives (*step 6: administer items to development sample*).

*Data used*. We used narrative assessments from three different sources in our UGME program: an integrative unit at the end of the preclinical curriculum at a Canadian University (source 1), a clinical clerkship in UGME (source 2), and a course to engage students in a reflective process to increase their awareness about their professional identity development (source 3). We favored different sources of narratives to increase the generalisability of our findings. The assessors that provided the narratives did not receive specific training on how to write good quality narratives, this is true for the three different data sources. Some assessments include scores and narratives, but only narratives were provided to us following ethical approval by our Local Institutional Review Board (2017–1494/St-Onge).

*Procedures*. Two team members (MC & VRD) independently applied the checklist (tool) to four series of narratives (Series 1: 150 narratives from source 1; Series 2: 100 narratives also from source 1; Series 3: 150 narratives from source 2, and Series 4: 150 narratives from source 3). These two team members met after each series to discuss document agreement, achieve a consensus on the presence/absence of each indicator and refine their understanding of the indicators. Interrater agreement (IA) was calculated after each of the four narrative rating rounds, using percentage of agreement. IA was calculated on the team members initial appraisal, that is, before consensus.

For Step 7 (*analysis)*, we calculated frequencies of occurrence for each quality indicator, per rater. We subsequently calculated IA (i.e., percentage of agreement) for each quality indicator to establish whether the team members arrived at the same decision regarding the presence or absence of a quality indicator for each narrative. Analyses were undertaken in SPSS (version 24).

Step 8 of DeVellis [16], is defined as *scale optimization*. We did not conduct this step since the list of indicators had been elaborated and rigorously refined in a published scoping review [10].

## Outcomes of Innovation

Six of the seven indicators identified in a published scoping review [10] were applied to the narratives. The indicator 5 “Provide justification for the mark/score given,” was not applicable since our data included the narratives but not the scores.

### Indicators’ frequencies of quality indicators of narratives

Overall frequencies (presence of an indicator) are presented per indicator in Table 1. The frequencies ranged from 0% to100%, showing that some quality indicators were more frequent than others. Table 1 signal that the most frequent indicators were “Describe performance with a focus on specific elements (attitudes, knowledge, skills)” (indicator 4), “Use language that is clear and easily understood” (indicator 6) and “Use a non-judgmental style” (indicator 7).

**Table 1:** Frequency measures of quality indicators of narratives.


	% OF OCCURRENCE OF INDICATOR PER RATER							

INDICATOR	SERIES 1: 150 NARRATIVES FROM SOURCE 1 (INTEGRATIVE UNIT)		SERIES 2: 100 NARRATIVES FROM SOURCE 1 (INTEGRATIVE UNIT)		SERIES 3: 150 NARRATIVES FROM SOURCE 2 (CLINICAL CLERKSHIP)		SERIES 4: 150 NARRATIVES FROM SOURCE 3 (COURSE ON REFLECTIVE PROCESS)	

1- Provides recommendations to students on how to improve their performance	Rater1	Rater2	Rater1	Rater2	Rater1	Rater2	Rater1	Rater2

	13.3%	10.7%	11.3%	11.3%	4%	4.7%	11.3%	10.7%

2- Provides a balanced message between positive elements and elements needing improvement	Rater1	Rater2	Rater1	Rater2	Rater1	Rater2	Rater1	Rater2

	13.3%	12.7%	13.3%	14.7%	3.3%	2.7%	14.7%	13.3%

3-Compares the observed performance to an expected standard performance	Rater1	Rater2	Rater1	Rater2	Rater1	Rater2	Rater1	Rater2

	0%	10.7%	19.3%	17.3%	5.3%	4.7%	27.3%	32%

4- Describe performance with a focus on specific elements (attitudes, knowledge, skills)	Rater1	Rater2	Rater1	Rater2	Rater1	Rater2	Rater1	Rater2

	82.7%	82.7%	78.7%	78.7%	92.7%	93.3%	100%	100%

5- Provide justification for the mark/score given	n/a	n/a	n/a	n/a	n/a	n/a	n/a	n/a

6- Use language that is clear and easily understood	Rater1	Rater2	Rater1	Rater2	Rater1	Rater2	Rater1	Rater2

	97.3%	99.3%	100%	99.3%	100%	100%	100%	100%

7- Use a non-judgmental style	Rater1	Rater2	Rater1	Rater2	Rater1	Rater2	Rater1	Rater2

	97.3%	99.3%	100%	100%	100%	100%	100%	100%


In Table 2 we present the quality score distribution per data set to investigate the range of quality in our data sets. Only six narratives received perfect quality scores. The lowest quality score was 2 (no narratives met none or only 1 quality indicator).

**Table 2:** Frequency measures of overall quality score per data set.


*6*	5 (2)	0 (0)	1 (0.7)

*5*	21 (8.4)	4 (2.6)	15 (10)

*4*	43 (17.2)	12 (8)	52 (34.7)

*3*	142 (56.8)	124 (82.7)	82 (54.7)

*2*	39 (15.6)	10 (6.7)	0 (0)

*1*	0 (0)	0 (0)	0 (0)

*0*	0 (0)	0 (0)	0 (0)


### Interrater agreement measures of quality indicators of narratives

IA ranged from 88.7% to 97.3% in the first series of narratives, from 94.7% to 100% in the second series, from 98% to 100% in the third series and from 95.3% to 100% in the fourth series of narratives. IA increased for indicator 4 “Describe performance with a focus on specific elements (attitudes, knowledge, skills)”, indicator 6 “Use language that is clear and easily understood” and indicator 7 “Use a non-judgmental style” from the first series of narratives to the last series of narratives which suggests an improvement in the standardization through its progression. IA mean per indicator ranged from 94.2% to 99.3%. Raters achieved the highest standardization for indicators: 4 (mean 97.5%), 6 (mean 99.0%) and 7 (mean 99.3%).

## Critical Reflection

Good quality narratives are essential to contribute to students’ learning and development, and to inform committees’ decisions about learners’ progression toward competence [3]. Given the call for strategies to document the quality of narrative assessment, our aim was to verify if we could apply, in a standardized manner, an evidence-informed list of quality indicators to narratives used in assessment. Our findings suggest that a standardized use of our checklist is possible. As such, the very practical outcome of this study is a checklist of quality indicators that can be used -by educators and administrators- to monitor the quality of narrative assessments when relying on them to make important decisions about students. Educators and administrators would only have to establish if a given indicator is present or not, avoiding the subjective appraisal of the quality of narratives.

Regarding the frequencies, we noted that some quality indicators were more frequent than others, such as “Use a language that is clear and easily understood” and “Use a non-judgemental style”. We assume that, in some ways, these two are probably the most intuitive indicators. On the other hand, some indicators were less frequently present in our sample of narratives. We noticed that assessors do not always link their comments to the required level of achievement relating to standards/expectations in their narratives. One plausible reason is that assessors may presume that the expected level of performance is already known by learners. Another important key point to consider is the live discussion that occurs (or not) between the assessor and the learner. A written comment may be formulated differently if the assessor has been able to discuss it with the learner. Likewise, assessors seem reluctant to provide recommendations to learners on how to improve their performance and to provide a balanced message between positive elements and elements needing improvement. This may be due to several issues such as the fear of damaging the relationship with students or lacking coaching abilities [6,10,17]. Indeed, previous studies of narratives have shown assessors’ tendencies for using positive comments rather than including critiques and recommendations for improvement [2,3,6]. Linguistic politeness strategies such as hedging are common in an attempt to avoid harming the student/supervisor relationship [17]. We also need to consider the purpose of the narrative: assessors might word it differently knowing, for example, that in addition to the student, a selection committee might see it. In other words, the purpose of the assessment and its confidential/non-confidential nature certainly influences not only the content of a narrative, but also the ways to articulate it.

Although our checklist seems suitable to appraise the quality of narratives, we must recognize that only six out of seven indicators were studied, as we did not have access to the scores associated with the sample of narratives. Empirical evidence regarding the application of the indicator 5 “Provide justification for the mark/score given” in a standardized way still needs to be addressed. Regarding this specific indicator, we recognize the usefulness of the “Quality of Assessment for Learning score” (QuAL score) [18], a tool has proven to be effective for qualitative comments which complete scores. Woods et al. [19] have also concluded that the tool can serve as a resource for faculty development. Notwithstanding, the QuAL score aims to evaluate short comments formulated in the context of workplace-based assessment, which is a very specific context.

Our checklist could be used as a tool by clinical supervisors during the process of writing narrative comments (to enhance their quality before or during writing narratives). Nevertheless, we also want to emphasize that a standardized use of our checklist does not exclude the fact that users (i.e., assessors or supervisors) would need training to be able to write good quality narratives. We recognize that the checklist has only been tested by members of the team, and therefore, a future step would be to test it as tool to write good quality narratives with assessors or supervisors after received training. While Nichols et al. [20] were unable to improve the quality of evaluators’ narratives by providing them with faculty development alone, others have demonstrated that it is possible to train faculty to provide higher quality narratives, primarily by focusing on improving narratives [21,22]. More recently, Mooney et al. [23] have demonstrated that a multipronged faculty development activity, including a broader perspective than traditional faculty development, can facilitate enhancement in the quality of narratives. Generating awareness of what constitute effective feedback may be a good starting point to improve written feedback [24]. A key finding from Nichols et al. [20] is the importance of applying techniques and strategies of deliberate practice during a faculty development session. Future studies could explore the possibility of such a workshop using our list to investigate if it has the potential to increase the quality of narratives provided by assessors.

## Disclaimer

The views expressed herein are those of the authors and do not necessarily reflect those of the Société des Médecins de l’Université de Sherbrooke.
